# A Conserved Aspartic Acid Is Important for Agonist (VUAA1) and Odorant/Tuning Receptor-Dependent Activation of the Insect Odorant Co-Receptor (Orco)

**DOI:** 10.1371/journal.pone.0070218

**Published:** 2013-07-23

**Authors:** Brijesh N. Kumar, Robert W. Taylor, Gregory M. Pask, Laurence J. Zwiebel, Richard D. Newcomb, David L. Christie

**Affiliations:** 1 School of Biological Sciences, University of Auckland, Auckland, New Zealand; 2 Department of Biological Sciences, Vanderbilt University, Nashville, Tennessee, United States of America; 3 Department of Pharmacology, Vanderbilt Brain Institute and Human Genetics Research, Institutes of Chemical Biology and Global Health and Program in Developmental Biology, Vanderbilt University Medical Centre, Nashville, Tennessee, United States of America; 4 Plant and Food Research, Auckland, New Zealand; Dalhousie University, Canada

## Abstract

Insect odorant receptors function as heteromeric odorant-gated cation channels comprising a conventional odorant-sensitive tuning receptor, and a conserved co-receptor (Orco). An Orco agonist, VUAA1, is able to activate both heteromeric and homomeric Orco-containing channels. Very little is known about specific residues in Orco that contribute to cation permeability and gating. We investigated the importance of two conserved Asp residues, one in each of transmembrane domains 5 and 7, for channel function by mutagenesis. *Drosophila melanogaster* Orco and its substitution mutants were expressed in HEK cells and VUAA1-stimulated channel activity was determined by Ca^2+^ influx and whole-cell patch clamp electrophysiology. Substitution of D466 in transmembrane 7 with amino acids other than glutamic acid resulted in a substantial reduction in channel activity. The D466E Orco substitution mutant was ∼2 times more sensitive to VUAA1. The permeability of the D466E Orco mutant to cations was unchanged relative to wild-type Orco. When D466E Orco is co-expressed with a conventional tuning odorant receptor, the heteromeric complex also shows increased sensitivity to an odorant. Thus, the effect of the D466E mutation is not specific to VUAA1 agonism or dependent on homomeric Orco assembly. We suggest the gain-of-activation characteristic of the D466E mutant identifies an amino acid that is likely to be important for activation of both heteromeric and homomeric insect odorant receptor channels.

## Introduction

Odorant receptors (Ors) are one of the main insect chemosensory receptor families required to sense olfactory cues in the environment [1]. They are seven transmembrane (TM) domain proteins with an inverted topology compared with G-protein coupled receptors [2]–[4]. Insect Ors form heteromeric complexes, of unknown stoichiometry, from a conventional, odorant-sensing (tuning) OR, and a co-receptor, now known as Orco [2], [5]. Insect Ors have been shown to function as odorant-gated non-selective cation channels [6], [7]. Metabotropic regulation of the channel may also occur but this remains unclear [1], [6], [8]–[10]. The conventional Ors are highly divergent and provide selectivity to a broad range of odorant compounds [11]. Their expression is restricted to specific olfactory receptor neurons (ORNs) [11]–[13]. In contrast, Orco is broadly expressed in ORNs and has not been shown to respond to odorants directly, but is essential for the response of Ors to odorants [14]. Orco is highly conserved across insect taxa, and *in vivo* is required for the trafficking of the Or complex to the dendritic membrane of ORNs [2], [15]. When heterologously expressed, Orco is capable of forming functional channels in the absence of a conventional receptor [6], [16]. These homomeric channels can be activated directly by the agonist VUAA1 [16] and its analogues [17]. Exploration of the structure activity relationships around the VUAA1 structure have identified several more potent agonists and additionally antagonists that are able to reduce both VUAA1- and odorant–evoked currents [17]–[19]. Studies with several heteromeric Or complexes indicate that the presence of an odorant-specific Or can alter the properties of the channel pore [20]–[22].

Relatively little is known about amino acid positions important for the gating and cation selectivity properties of Orco channels. Modification of a TVGYG sequence in TM6 of *Drosophila melanogaster* (DmelOrco) reduced K^+^ permeability [6] and a Y464A mutation in TM7 of the *Bombyx mori* Orco (BmOrco) in combination with BmOr-1 results in a small increase in K^+^ selectivity [20]. As conserved acidic amino acids are known to have a role in ion permeation and gating of cation channels [23]–[25], we have investigated the importance of conserved Asp residues in predicted TMs 5 and 7 of DmelOrco. Here we observe that the Asp residue associated with Orco TM7 is linked to channel activation, where substitution of a glutamic acid at this position gave rise to an Orco variant that is more sensitive to both VUAA1 and odorant agonism.

## Materials and Methods

### Chemicals

VUAA1 (N-(4-ethylphenyl)-2-((4-ethyl-5-(3-pyridinyl)-4H-1, 2, 4-triazol-3-yl)thio)acetamide) was purchased from Interbioscreen Ltd (ID# STOC3S-70586) or from ChemBridge corporation (ID# 7116565). Eugenol (CAS 97-53-0) and methyl hexanoate (CAS 106-70-7) were purchased from Sigma. All compounds were first dissolved in DMSO and subsequently diluted into the appropriate buffer solution.

### Orco and OR Plasmids

DmelOrco in pcDNA3.1+ was modified to include an N-terminal myc epitope (EQKLISEEDL) by PCR. N-myc DmelOrco was transferred into pcDNA5/FRT/TO using *Kpn*I and *Not*1 sites. Constructs encoding the D357N, D466N and D466E variants were produced by GenScript USA Inc. using the WT N-myc DmelOrco plasmid as template. DmelOR22a and *Anopheles gambiae* (AgOR65) were cloned into pCI (Promega).

### Cell Culture, Transfection and Ca^2+^ Imaging

Flp-In 293 T-REX cells (Invitrogen) were grown in Dulbecco’s modified Eagle’s medium (Invitrogen) supplemented with 10% fetal bovine serum (Qualified, New Zealand Origin, Invitrogen #1009148), 4 mM glutamine, blasticidin (10 µg/ml) and zeocin (100 µg/ml). To make stable cell lines Flp-In 293 T-REX cells (700,000 cells/well in 6 well plates) were transfected with 2 µg of total plasmid DNA (pcDNA5/FRT/TO-N-myc DmelOrco: pOG44 FLP recombinase plasmid, Invitrogen); 9∶1 ratio and 10 µl of Lipofectamine 2000 (Invitrogen). Two days later, cells were trypsinized, diluted, plated in 10 cm dishes and selected with medium containing blasticidin and hygromycin B (Invitrogen, 200 µg/ml).

Two alternative methods were used to determine changes in intracellular Ca^2+^. In the first, cells were plated (50,000 cells/well) in 96-well clear bottom, black-walled plates (BD Biocoat Cat. #356640). After 1 day, cells were treated with 0.1 µg/ml tetracycline to induce Orco expression for 24 h. The medium was then removed and the cells loaded (30 min at 37°C, followed by 1 h at room temperature) with Fluo-4 NW (Invitrogen) prepared as suggested by the manufacturer in Hank’s buffer containing Ca^2+^ and Mg^2+^. Ca^2+^ fluorescence was measured in an Envision multilabel plate reader (Perkin Elmer). The following settings were used: excitation filter, FITC 485 nm; emission filter, 520 nm; bottom-fitted dichroic mirror, FITC 505; bottom excitation, bottom sensor; measurement distance: 6.5 mm. Fluorescence readings were taken every 0.4 seconds; agonist, freshly-diluted from DMSO stocks into Hank’s buffer, was injected automatically after 8 seconds. Calcium fluorescence experiments were also carried out with cells plated (20,000 cells/well) in 364-well cell culture plates (Greiner) and loaded with Fluo-4 AM (Molecular Probes). These cells were transfected with either *Drosophila melanogaster* Or22a (DmOr22a) or *Anopheles gambiae* OR65 (AgOr65). Calcium was assayed in an FDSS6000 plate reader (Hammamatsu) as described [26]. Curve generation and statistical analysis was performed using Prism software (GraphPad).

### Cell-surface Biotinylation and SDS Polyacrylamide Gel Electrophoresis

Flp-In 293 T-REX cell lines expressing DmelOrco and its variants were plated (700,000 cells/well in a 6-well plate, grown for 24 h, and induced with tetracycline (0.1 µg/ml) for 24h and proteins at the cell-surface labeled with a membrane impermeable biotinylation reagent. The procedure for biotinylation was similar to that used previously for membrane transporters [27]. Briefly, the cells were washed with PBS and labeled with, EZ-Link-Sulfo-NHS-SS-Biotin (ThermoScience). The reagent was removed, and the cells washed and lysed in 20 mM Tris, 137 mM NaCl, 1 mM EDTA, pH 7.6 buffer, containing 1% Triton X-100, 1% sodium deoxycholate, 0.1% SDS, and protease inhibitors (Complete™ Mini protease inhibitor mixture, Roche Molecular Biochemicals). The lysate was obtained by centrifugation. A sample of the lysate was used for the purification of biotinylated proteins using Neutravidin beads (ThermoScience). Following incubation, the beads were recovered by centrifugation and washed to remove non-specifically bound proteins. The beads were resuspended with an equal volume of 2×SDS reducing buffer (125 mM Tris, 4% SDS, 20% glycerol, and 10% β-mercaptoethanol) and incubated for1 h at 37°C. Aliquots of the lysate samples and biotinylated fraction were run on 10% SDS polyacrylamide gels and subjected to Western blotting [27]. The blot was probed with mouse anti-myc antibodies (Santa Cruz, sc-40) followed by goat-anti-mouse horseradish peroxidase conjugate (Bio Rad Cat. #170–6516). Chemiluminescence was detected using a Fuji LAS-1000 digital imaging system.

### Patch-Clamp Recording of HEK Cells

Currents from OR-expressing HEK293 cells were recorded as previously demonstrated [16]. Electrodes were filled with internal solution [120 mM KCl, 30 mM D-glucose, 10 mM Hepes, 2 mM MgCl_2_, 1.1 mM EGTA, and 0.1 CaCl_2_ (pH 7.35, 280 mOsm)]. External (bath) solution contained 130 mM NaCl, 34 mM D-glucose, 10 mM Hepes, 1.5 mM CaCl_2_, 1.3 mM KH_2_PO_4_, and 0.5 mM MgSO_4_ (pH 7.35, 300 mOsm). Compounds were diluted in external solution and locally perfused to the cell using a Perfusion Pencil (Automate Scientific). Whole-cell recordings were sampled at 10 kHz and filtered at 5 kHz. Cation permeability assays and analysis were performed as previously described [22].

## Results

### A conserved Asp Residue in TM 7 of Orco is Important for Channel Activity

**Figure 1:**
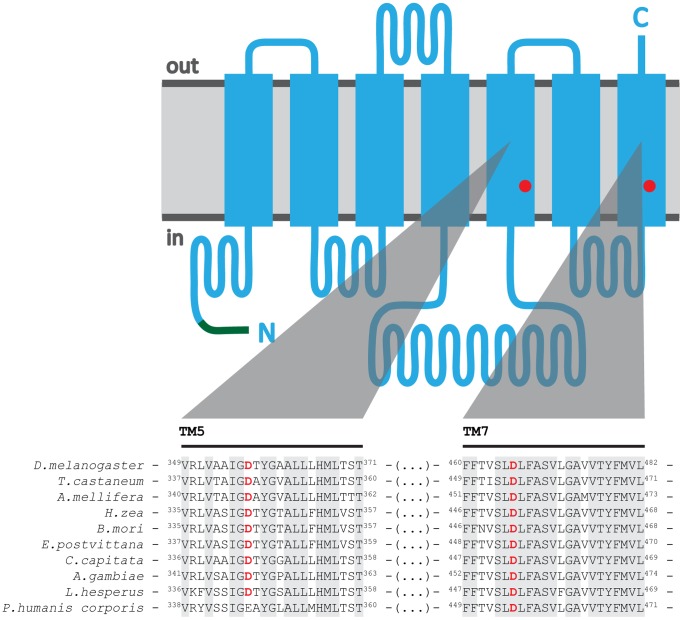
Schematic diagram of DmelOrco showing aspartic acid residues present in TM domains 5 and 7 that are highly conserved in other insect species. The TM domains and intracellular and extracellular regions of Dmel Orco were predicted using TMHMM [28], [29]. The green line indicates the presence of a myc-epitope at the N-terminus used to detect Orco by western blotting; to simplify comparison with the other Orco sequences we have retained the numbering of the WT DmelOrco protein. The red dots show Asp residues at positions 357 and 466 in TM5 and TM 7. The DmelOrco sequence was aligned using ClustalW with Orco sequences from various other species representing 6 insect orders. Sequences corresponding to TMs 5 and 7 are aligned; conserved Asp residues are shown in red. The Asp residue at position 357 is highly conserved; only the *P. humus corporis* sequence showed a difference; Asp is replaced by Glu. An Asp residue equivalent to D466 in DmelOrco is found in all sequences. The light gray shading shows absolutely conserved regions in TMs 5 and 7.The accession numbers for the Orco protein sequences are: *Tribolium castaneum*, EFA05687; *Apis mellifera*, NP_001128415; *Helicoverpa zea*, AAX14773; *Bombyx mori*, NP_001037060; *Epiphyas postvittana*, ACJ12928;*Ceratitis capitata*, AAX14775.1; *Drosophila melanogaster*, NP_524235; *Anopheles gambiae*, AAX14774; *Lygus Hesperus*, AFX73447; and *Pediculus humanus corporis*, EEB12924.

**Figure 2:**
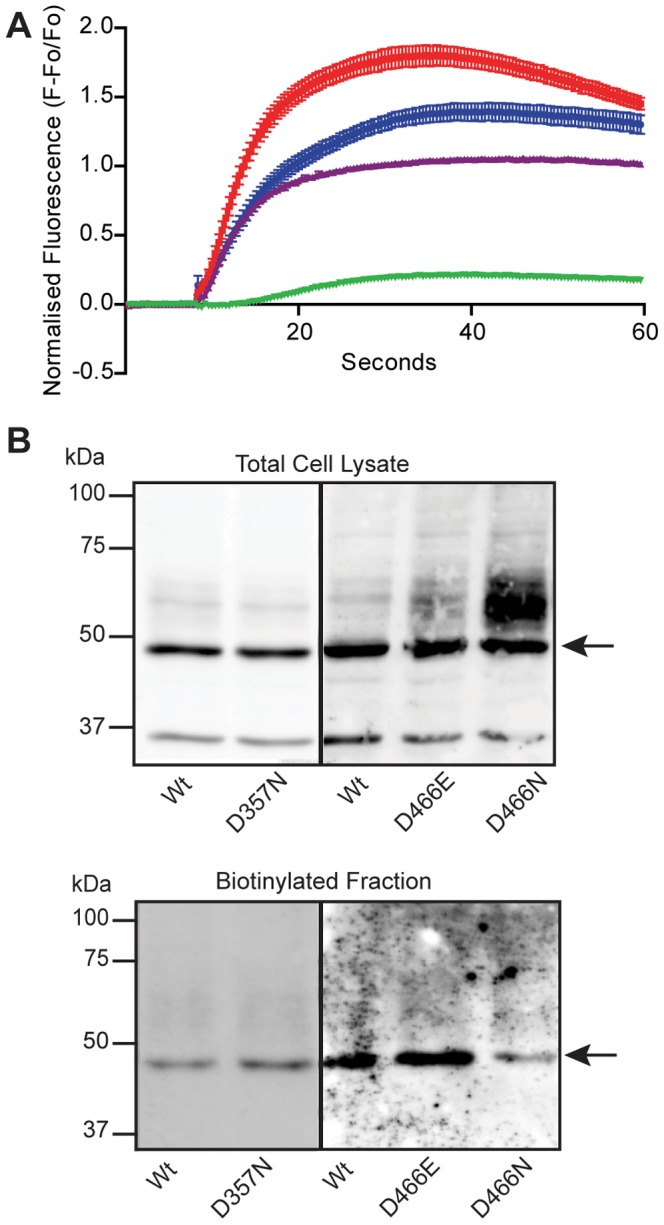
Effect of amino acid substitution of D357 and D466 in DmelOrco on VUAA1-stimulated Ca^2+^ influx activity and protein expression. (A) The rate of Ca^2+^ influx stimulated by 100 µM VUAA1 of FlpIn 293-T-REX cells expressing D466E (red), WT (blue), D357N (purple) and D466N (Green) Orco (the data represent the means ± SEM of 6 replicates). (B) Comparison of the relative protein expression of Orco and its D357, D466E and D466N variants in FlpIn 293-T-REX cells. Samples of whole-cell lysates and the biotinylated fraction (cell-surface) were analysed by western blotting with myc antibodies. The comparison of Orco with D357, and Orco with D466E and D466N, are from two separate experiments. The positions of molecular mass marker proteins are indicated. The arrow indicates the band expected to correspond to Orco.

Fig. 1 shows the membrane topology of *Drosophila melanogaster* (DmelOrco) as predicted using TMHMM [28], [29]. Transmembrane domains 5 and 7 each contain an Asp residue that is highly conserved in Orco from other species. The importance of both conserved Asp residues for channel function in Orco was investigated by mutagenesis and functional characterization using Ca^2+^ mobilization assays. Substitution mutants of D357 and D466 were stably expressed in FlpIn 293 T-Rex cells and stimulated with VUAA1. The Ca^2+^ influx kinetics of WT DmelOrco and its D357N, D466N and D466E substitution mutants following addition of agonist (100 µM VUAA1) are shown in Fig. 2A. The D357N mutant retained ∼78% of the activity seen with WT Orco, indicating an Asp at this position is not essential. In contrast, the D466N mutant showed greatly reduced activity (∼15% of WT Orco). When D466 was replaced with a range of other amino acids, including Ser, Ala and Cys, none of these mutants exhibited significant activity (data not shown). It is therefore likely that an Asp, or an acidic amino acid, at position 466 is critical for DmelOrco function. Interestingly when D466 was replaced with a glutamic acid, cells expressing the D466E mutant not only retained activity, but also showed an apparent increase in the activation levels compared with WT Orco (Fig. 2A).

We investigated whether differences in the activity levels of Orco and its variants were due to changes in protein expression. The expression of Orco and its variants was compared in both whole-cell lysates, and purified biotinylated (cell-surface), fractions by western blotting with myc-antibodies (Fig. 2B). The comparison of Orco with D357, and with D466E and D466N, are from two different experiments. In cell lysates and biotinylated fractions the level of expression of Orco was similar to its variants (∼50 kDa band) except for D466N. The ∼50 kDa band was reduced in D466N lysates; this coincided with the appearance of a broad higher molecular weight band that may correspond to aggregated forms of Orco. The level of the ∼50 kDa component was also decreased in the biotinylated fraction of D466N compared to both Orco and D466E (Fig. 2B lower). Thus it appears the decreased activity seen for the D466N variant may, at least in part, be due to reduced expression at the cell surface. The increased response of the D466E variant to VUAA1 does not appear to be explained by higher total or cell-surface expression.

### D466E Orco is More Sensitive to Agonist Activation by VUAA1 than Wild-type Orco

**Figure 3:**
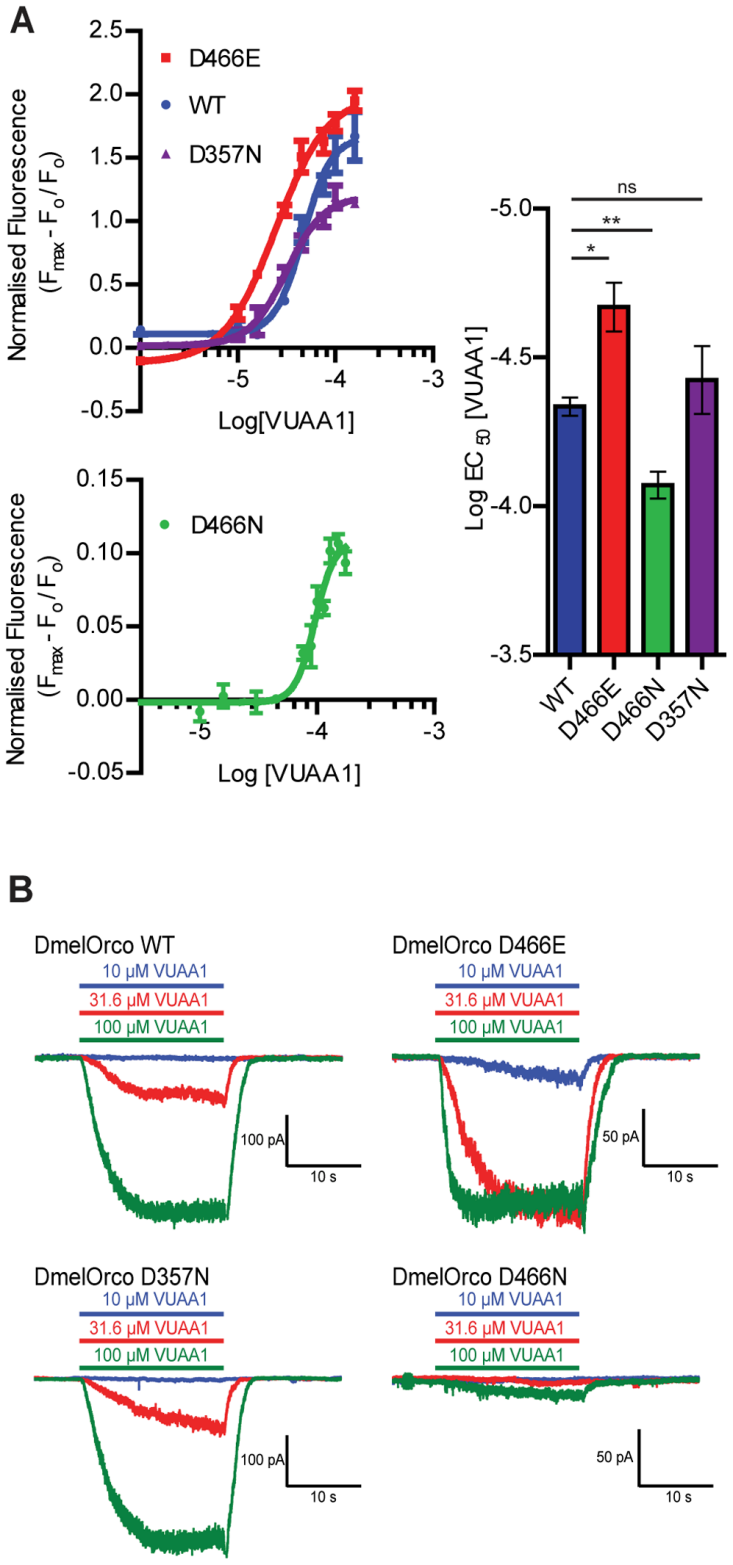
D466E Orco is activated more rapidly and is more sensitive to VUAA1 than WT Orco. (A) Concentration-response curves for the effect of VUAA1 on WT, D466E, D466N, and D357N Orco as determined by Ca^2+^ imaging. The D466E variant is significantly more sensitive to VUAA1 (LogEC_50_ = −4.67±0.08) than WT DmelOrco (LogEC_50_ = −4.33±0.03) (*p<0.05, n = 3), and the D466N variant (LogEC_50_ = −4.07±0.05) is significantly less sensitive (**p<0.01, n = 3). The sensitivity of the D357N (LogEC_50_ = −4.42±0.11) variant does not differ significantly from WT. (B) Representative traces of evoked currents in FlpIn 293 TREX cells expressing WT, D466E, D357N and D466N DmelOrco to 10, 31.6 and 100 µM VUAA1.

The experiments above indicate cells expressing the D466E mutant not only retain activity, but appear to be more responsive to VUAA1 (Fig. 2A). Dose response curves for VUAA1-stimulated Ca^2+^ influx of cells expressing D466E, D466N, D357N and DmelOrco WT are shown in Fig. 3A. Here, the response of the D466E mutant to VUAA1 exhibits a clear leftward shift in potency, indicating that this variant is ∼ 2 times more sensitive to VUAA1 (LogEC_50_ = −4.67±0.08) than WT Orco (LogEC_50_ = −4.33±0.03). This potency shift was not observed in D357N cells, which had comparable VUAA1 sensitivity to WT (LogEC_50_ = −4.42±0.11). The dose response curve for D466N is shown separately as the activity levels were much lower. This mutant is significantly less sensitive to VUAA1 (LogEC_50_ = −4.07±0.05) than WT Orco.

The response of cells expressing D466E, D466N, D357N and DmelOrco WT to different concentrations of VUAA1 was also assessed by whole-cell patch clamp experiments (Fig. 3B). A similar inward current response to 100 µM VUAA1 was seen for WT, D466E and D357N Orco. A very small current response is seen for D466N. The inward current response of D466E to 30 µM VUAA1 indicates this mutant is more sensitive to VUAA1; it is also the only Orco variant to show a current response to stimulation with 10 µM VUAA1 (Fig. 3B).

### D466E Orco Displays Similar Cation Permeability to WT Orco

**Figure 4:**
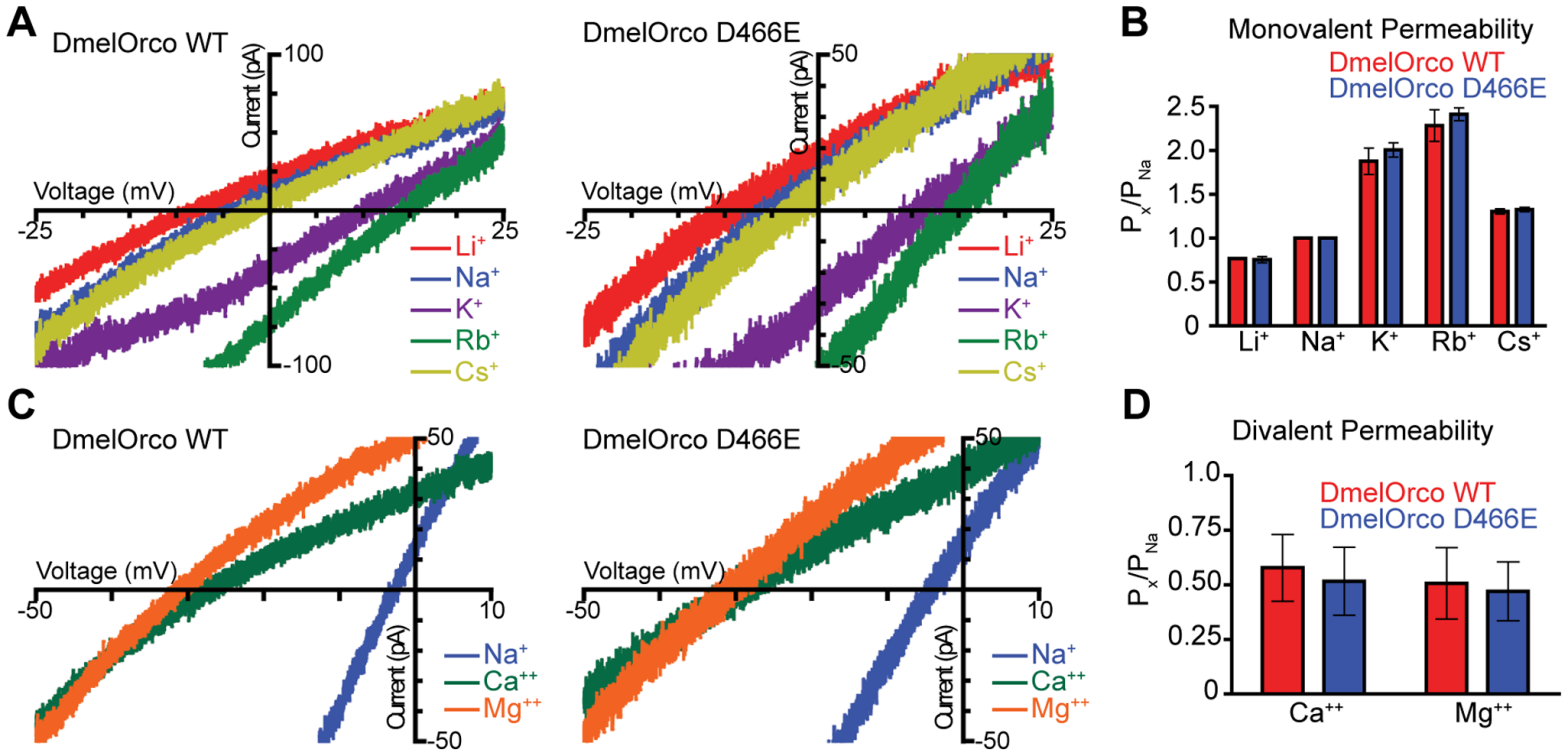
The D466E mutation does not affect the monovalent or divalent cation permeability of the Orco channel. Representative VUAA1-currents of WT and D466E Orco in extracellular solutions containing 150 mM of the indicated monovalent (A) or divalent cation (C) and 100 µM VUAA1. (B,D) Histograms of the relative permeability of monovalent and divalent cations through WT and D466E Orco complexes relative to Na^+^. A two-way ANOVA and a Bonferroni post test found no significant differences between WT and D466E (p>0.05, n = 4).

These data are consistent with a hypothesis wherein the D466E Orco variant is able to activate more efficiently in response to the Orco agonist VUAA1. Because Orco channels are permeable to both monovalent and divalent cations [6], we also characterized the relative cation permeability of D466E mutant channels as compared to WT Orco using whole-cell patch clamp electrophysiology (Fig. 4A–D). Here, the relative permeability to monovalent cations was determined by subjecting steady-state VUAA1-induced currents to a voltage ramp to determine the reversal potential, where net current through the channel is zero, in extracellular solutions containing a single cation (Fig. 4A,B). Both D466E and WT DmelOrco channels showed no difference in monovalent cation permeability, and the relative permeability sequence of Rb^+^>K^+^>Cs^+^>Na^+^>Li^+^ (Eisenmann III) is consistent with a previous studies of Orco [22]. The relative permeability of D466E and WT DmelOrco channels to Ca^2+^ and Mg^2+^ were also tested with extracellular solutions containing a single divalent cation (Fig. 4C,D). Once again, no differences were seen in the permeability of D466E and WT DmelOrco proteins to Ca^2+^ and Mg^2+^.

### D466E Orco is More Sensitive to Activation by an Odorant in the Presence of a Conventional Or

**Figure 5:**
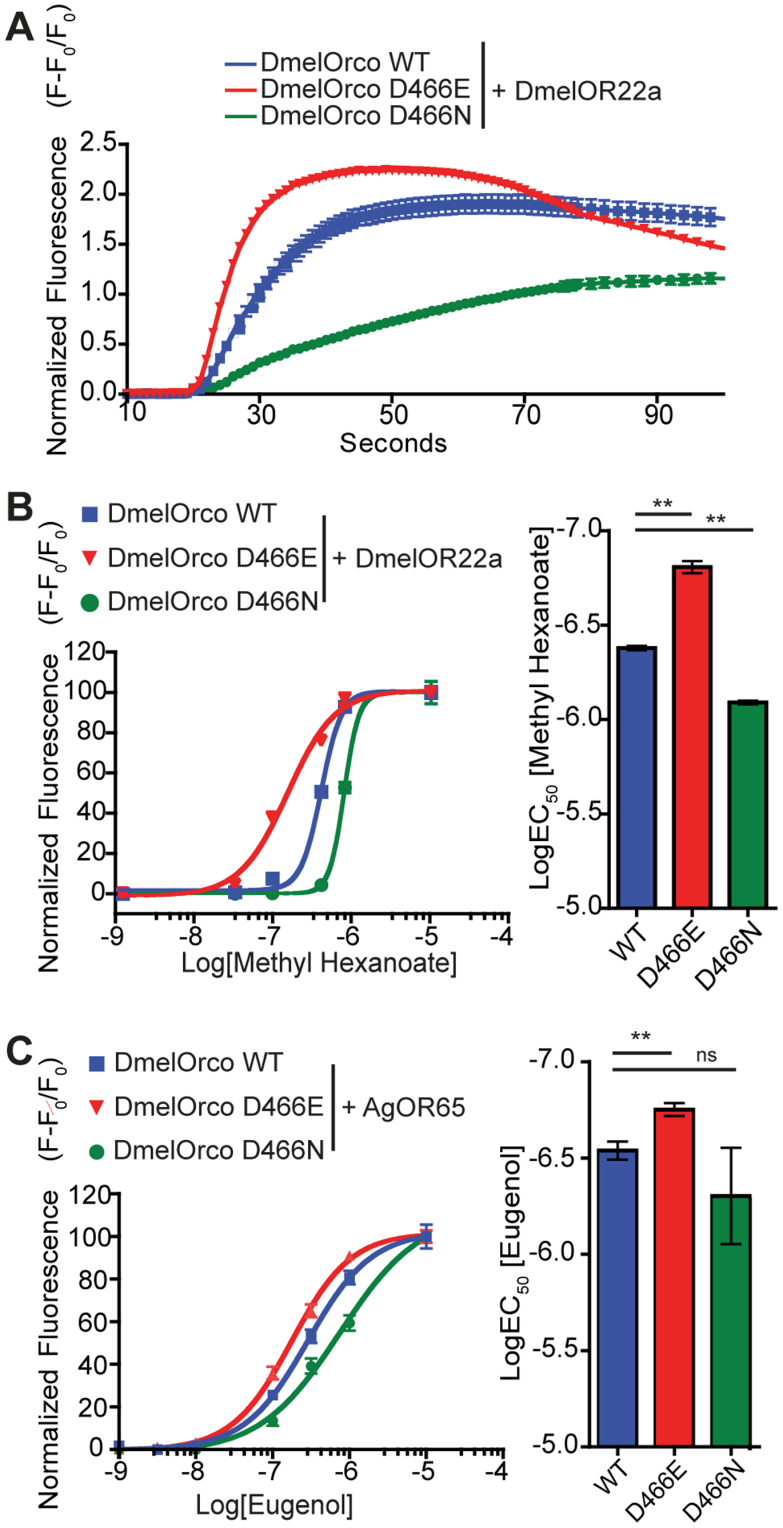
D466E Orco is more sensitive to odorants when expressed together with a tuning Or. (A,B) Flp-In 293 T-REX cells stably transfected with WT, D476E and D476N DmelOrco were transfected with pCI-DmelOr22a. Orco was induced with tetracycline (0.3 µg/ml) and calcium fluorescence determined before and after the addition of methyl hexanaoate. (A) Time-course of calcium fluorescence following addition of 10 µM methyl hexanaote. (B) Concentration-response curves of WT D466E and D466N DmelOrco: DmelOR22a complexes to methyl hexanaote. LogEC_50_ values for WT (−6.38±0.018) and D466E (−6.81±0.03) differed significantly (**p<0.001), as did WT and D466N (−6.09±0.01). (C) Concentration-response curves of WT, D466E, and D466N DmelOrco cells transfected with AgOR65 to eugenol. LogEC_50_ values for WT (−6.54±0.05) and D466E (−6.75±0.03) differed significantly (**p<0.001), while D466N (−6.34±0.04) did not.

It was important to know whether the increased sensitivity to VUAA1 observed for D466E extended to odorant activation of heteromeric Or complexes. To investigate this, cells expressing D466E, D466N and WT DmelOrco were transiently transfected with the tuning receptor DmelOR22a and Ca^2+^ influx determined in response to increasing concentrations of methyl hexanoate (Fig. 5A,B). In these studies D466E Orco/DmelOR22a complexes were significantly more sensitive to activation by methyl hexanoate (LogEC_50_ = −6.81±0.03) than WT Orco complexes (LogEC_50_ = −6.38±0.01), while D466N Orco complexes (LogEC_50_ = −6.09±0.01) were significantly less sensitive to activation. Essentially identical responses were observed to ethyl hexanoate, another odorant known to elicit DmOR22a/Orco-dependent responses (data not shown).

Conventional tuning OR subunits have been shown to form functional complexes with Orco channels from a range of insects [15], [21], [30], [31]. To further demonstrate the changes in sensitivity to odorant agonism, the Or65 subunit from the malaria mosquito *Anopheles gambiae* (AgOR65) was transfected into cell lines stably expressing D466E, D466N and WT DmelOrco and stimulated with the AgOR65 agonist, eugenol [12]. In these studies, the D466E Orco/AgOR65 combination (LogEC_50_ = −6.75±0.03) is similarly more sensitive to eugenol activation than WT Orco complexes (LogEC_50_ = −6.54±0.05). The difference between the sensitivity of D466N Orco (LogEC_50_ = −6.34±0.04) and WT Orco complexes is not significant.

## Discussion

We first studied the effects of substitution of conserved Asp residues in TM5 and TM7 on the channel properties of Orco in the absence of a conventional tuning Or. This approach is feasible due to the ability of Orco to form functional homomeric cation channels [6] and the availability of VUAA1, an allosteric Orco agonist [16]. Use of homomeric channels facilitates the interpretation of the effects of a specific mutation in Orco, avoiding complications of possible altered interactions between Orco and a tuning receptor, or difficulties in interpreting results due to the presence of mixtures of both homomeric and heteromeric Orco complexes. Our studies identify the importance of the conserved acidic amino acid in TM7 (Asp466 in WT DmelOrco) for channel activation.

All experiments were carried out with FlpIn 293 T-REX cells lines stably expressing N-myc tagged DmelOrco and its substitution mutants. These lines provide consistent expression of Orco following induction with tetracycline and are suitable for functional assays based on both higher throughput Ca^2+^ influx assays and patch-clamp electrophysiology. Biotinylation studies indicated that cell-surface expression of all of the substitution variants was similar to WT DmelOrco with the exception of D466N. Western blotting of lysates expressing this mutant showed lower levels of the expected ∼50 kDa Orco band, and increased amounts of a higher molecular weight component which may represent aggregated material resulting from less efficient folding and processing of this variant in the endoplasmic reticulum. The low levels of functional activity made it difficult to fully characterize the D466N mutant. The experiments in Figs 2B and 3A, however, suggest that impaired activation, as well as reduced cell-surface expression, may be responsible for the low levels of channel responses seen for this mutant.

Key features of ion channel proteins are regions that regulate ion-permeability and gating. Currently there is no structure available for Orco, and the lack of similarity of Orco to other, more characterized cation channel families precludes the use of homology-based modeling approaches. Mutagenesis experiments have indicated that regions of TM6 of DmelOrco and TM7 of *B. mori* Orco (BmOrco) affect K^+^ selectivity [6], [20]. The main finding of the present study is that the D466 position is likely to play a critical role in Orco activation. Of the several D466 substitution variants examined in this study, only D466E displayed significant responses to VUAA1 stimulation. The importance of this residue is further supported by the observation that D466E variant channels are more sensitive in the response to both a direct activator of Orco (VUAA1), and conventional Or-mediated ligands (methyl hexanoate and eugenol). This may be the result of the inductive effect of additional carbon in the glutamic acid R-group that gives rise to significantly higher pK_a_ than aspartic acid, or the extra carbon could simply allow for greater flexibility that might have a role in channel gating. The D466E mutation in our study (D466E in WT DmelOrco) is located 12 amino acids nearer to the predicted cytoplasmic end of TM7 than the position of the Y478A mutation found to increase BmOrco K^+^ permeability [20]. Our results suggest that D466 is important for channel activation, but does not affect cation permeability. A similar gain-of-activation function was seen whether D466E was stimulated by VUAA1 or an odorant in the presence of conventional Or from the same or a distinct species. This indicates that the effect is not specific to allosteric (VUAA1) agonism, nor is it limited to homomeric Orco complexes. The activation seen with D466E is consistent with this position being important for channel gating. Recently an Orco antagonist, VU0183254 has been shown to affect both VUAA1-stimulated and Or-stimulated odorant responses leading to the suggestion that binding of the antagonist ‘locks’ the channel shut [19]. This antagonist inhibited VUAA1-stimulated Ca^2+^ influx of both WT and D466E Orco with similar potency (data not shown) suggesting position D466 does not affect antagonist binding. We suggest that the D466E mutant has a conformational state that favors the opening of the channel gate. An aspartic acid residue (Asp^433^) in the closing gate of the acid-sensing ion channel ASIC1 has been shown to determine the stability of the open state [23].

In summary, we provide evidence based on the gain-of-activation seen for the D466E mutant that a residue equivalent to D466 in TM7 of DmelOrco may be involved in gating of the channel. The fact that the D466E mutants respond more sensitively to both direct agonism and odorants in the presence of a tuning Or suggests that both activation mechanisms induce similar conformational changes leading to opening of the channel to cations.
